# The Effect of Behavioural Indicators of Calf Discomfort Following Routine Procedures on Cow Maternal Care

**DOI:** 10.3390/ani10010087

**Published:** 2020-01-06

**Authors:** Simon P. Turner, Kirstin McIlvaney, Jo Donbavand, Matt J. Turner

**Affiliations:** 1 SRUC (Scotland’s Rural College), Kings Buildings, West Mains Road, Edinburgh EH9 3JG, UK; jo.donbavand@sruc.ac.uk (J.D.); Matt.Turner@sainsburys.co.uk (M.J.T.); 2 The Royal (Dick) School of Veterinary Studies, University of Edinburgh, Easter Bush, Edinburgh EH9 3JG, UK; Kirstin.mcilvaney@ed.ac.uk

**Keywords:** maternal behaviour, cattle, castration, pain, empathy

## Abstract

**Simple Summary:**

Animals are believed to show empathy when witnessing others in pain. Painful experiences alter arousal and induce specific forms of behaviour to reduce pain. It is unclear if observers respond simply to the level of arousal or can detect differences in the expression of pain indicators. We explored whether beef cows adjust their care-giving behaviours dependent upon their calf’s expression of pain following ear tagging and (in males) castration. Greatest empathy is expected when witnessing aversive experiences in kin, and beef cows show vigorous maternal care shortly after calving. We therefore hypothesise that cows show increased maternal care towards calves that display the most behavioural evidence of pain. Observations were made for 20 min before and 30 min after tagging (and castration) of 58 calves. Increased arousal in the calf was associated with increased maternal care behaviour by the cow. However, the expression of pain indicator behaviours did not influence the cow’s behaviour. Males that had experienced castration did not receive more care than females. Cows seem to be insensitive to behaviours of their calf previously shown to indicate pain but this may be due to the subtlety of behavioural expression in young calves or the recent experience of calving.

**Abstract:**

Pain causes altered arousal and specific behaviours that are rare at other times (pain indicators). We examined whether beef cows (mean age 6 years) are sensitive to pain indicators in addition to arousal following ear tagging and rubber ring castration (males only, *n* = 31) of their calf (*n* = 58). Behavioural pain indicators and activity were recorded continuously for 20 min before and 2 h after handling. The 30 min period posthandling captured the peak of behavioural change during this window. Cow maternal and maintenance behaviours were recorded for 20 min before and for 30 min after calf handling. Principal component (PC) analysis identified two dimensions (48% of the variance) in calf behaviour. Arousal and two active pain indicators loaded onto PC1 were shown by linear mixed models to positively affect some maternal behaviours. Lethargy, passive pain indicators and further active pain indicators loaded onto PC2 did not affect cow behaviour. Castration did not result in male calves receiving more maternal attention. Cows alter maternal behaviour in response to calf activity but not behaviours previously shown to indicate pain. Maternal care peaks soon after calving, but recent calving stress or the subtlety of neonatal behavioural expression may constrain cows in responding to the individual behavioural expression of their calf. Future studies exploring empathy should be aware of the constraints on behavioural expression associated with the neonatal and periparturient period.

## 1. Introduction

Empathy describes an emotional reaction when observing the experiences of another [1]. The ability of nonhuman primates to show emotional empathetic responses has been extensively studied [2]. Less is known about the capacity of other species to experience emotional distress when witnessing the experiences of conspecifics (see [2] for a review).

Some species have the capacity to discriminate between facial expressions or other cues induced in conspecifics by situations of contrasting emotional valence (rats, mice, sheep, goats; [3,4,5,6]). Other studies suggest that an ability to discriminate between distressed and non-distressed conspecifics does not extend to an alteration in emotional state of the observer (e.g., pigs, lambs, hens; [7,8,9]). However, greatest emotional empathy is expected to be shown when witnessing emotional responses of kin due to potential fitness benefits [10,11]. Therefore, if non-primate species show empathy, it is likely to be detected most readily when observing the distress of close relatives at a vulnerable age. Indeed, Edgar et al. (2010), Hild et al. (2011) and Futro et al. (2015) [12,13,14] have all shown that mother ewes are capable of differentiating between a lamb that had experienced handling stress and one that had received handling stress and painful procedures. In some of these studies, the maternal attention directed towards the lamb was correlated with the amount of active pain behaviour shown by the lamb. Similarly, rats direct more attention to pups that had received a painful procedure as compared to pups that had simply been handled [3]. Even though these studies cannot confirm the existence of emotional perspective taking, they indicate that mothers in these species are capable of distinguishing between offspring that had been handled from those that had been handled and were in pain, and altering their behaviour accordingly.

However, experience of pain is likely to alter both the level of arousal of the subject and the occurrence of behaviours designed to alleviate pain or solicit assistance from conspecifics [15]. It is unclear from previous work how responsive mothers are to specific indicators of pain separate from general arousal. Earlier work has shown that mothers are indeed responsive to the level of arousal of their young even when these are not in pain. For example, Futro et al. (2015) [14] found that ewes direct greater attention to a lamb that was handled but did not receive any painful procedures as compared to a littermate that was not handled, suggesting that the period of isolation or altered behaviour upon return affected the ewe’s behaviour even in the absence of offspring pain. It is important to quantify the effect of the subject’s activity level on the observer’s response in order to determine that the observer is sensitive to behaviours that have been validated as specific indicators of pain in addition to simply heightened activity.

In the observational study described here we recorded the reaction of beef cows to the ear tagging (females) and simultaneous tagging and rubber ring castration (males) of their calves. We hypothesised that cows would show increased maternal care towards male calves which had also experienced castration (hypothesis 1) and that individual differences in the amount of pain behaviours shown by the calf, separate from its level of activity, would affect the care-giving response of the cow (hypothesis 2).

## 2. Methods

### 2.1. Ethical Note

The study was approved by the SRUC Animal Experiments Committee (application ED/AE/16 of 2013). All observations were made during routine handling of calves during farm procedures and no calves were ear tagged or castrated as a requirement of this study. No calves required subsequent veterinary attention following ear tagging or castration.

### 2.2. Overview

The natural variation in calf activity and behaviours identified by previous studies to be indicative of pain (e.g., [15]) were both recorded following ear tagging and (in males) castration. Calves were used as their own controls by considering the change in behavioural expression pre- to posthandling. Principle component analysis was then used to identify latent variables that best described calf responses based upon this wide range of behaviours. The association between the calf’s score on these behavioural dimensions and its mother’s response were tested.

### 2.3. Animals

The study was performed at the SRUC Beef Research Centre using 58 crossbred Aberdeen Angus × Limousin cows with single calves (31 males, 27 females). Cows and calves were not genotyped as the sample size and structure did not allow robust genetic analysis of behavioural responses. Cows were a mean of 6 years of age (SD 3.1) at the time of the study. The sires of the calves were crossbred Limousin (*n* = 21), crossbred Aberdeen Angus (*n* = 29), Luing (*n* = 8) or Charolais (*n* = 9). All calves were spring born over a 10 week period in the same calving season and had experienced unassisted births. Each cow/calf pair was housed in a single deep-straw bedded pen (approximately 12 m × 6 m) with cattle visible in the neighbouring pens through a barred division. Cows and calves were able to see the behaviour of animals in adjacent pens. This represents normal commercial practice in the UK and was preferable to visually isolating cows which would likely have caused fear or stress that would have varied in extent between individual cows and may have masked their response to distress in the calf. However, a minimum 4 h period separated handling of calves in adjacent pens. Whilst cows may have reacted to observing behaviour in the adjacent pen, the cow’s own behaviour was used as a baseline with which to compare her behaviour after handling of her own calf. Therefore, if the cow’s behaviour was influenced by witnessing the behaviour of others in the adjacent pen over 4 h earlier, this would have been accounted for in the approach we used.

### 2.4. Handling

Handling of newborn calves occurred at an estimated median of 14.5 (interquartile range 7–24) hours after birth at a mean weight of 44 (SD 6.9) kg and was performed by the same experienced stockperson throughout. Prior to handling, humans had not entered the pen for a median of 3.1 (interquartile range 2.0–6.0) hours. The handler entered the pen, walked the calf to the front of the pen, weighed the calf using a portable calf weigh crate, and then manually restrained it whilst inserting an ear tag into each ear and lastly applying a rubber ring to the testes and scrotum of males. No anaesthetic or analgesic was used in line with standard commercial practice in the UK. Procedures were always performed in the same order and the handler was in the pen for a median of 2.0 (interquartile range 2.0–3.25) minutes. Visual and auditory contact between the cow and calf was possible throughout and the cow remained unrestrained during handling of her calf. The mean ambient temperature recorded at the time of handling was 9.7 (SD 4.79) °C.

### 2.5. Behavioural Observations

#### 2.5.1. Immediate Response to Handling

All live behavioural observations were performed by the same single observer throughout. The vigour of the calf’s initial response to treatment was recorded immediately upon insertion of the ear tags and, for males, application of the rubber ring. As two procedures were completed for females (application of two tags) and three for males (two tags and the rubber ring), the strongest response was taken as the score for each animal. An adaptation of the scale developed by [16] was used whereby 0 = no response; 1 = mild struggling (movement of head or steps forward/backwards); 2 = actively trying to escape or the handler needs to use force to restrain; 3 = vigorously struggling, knocks handler off balance.

Cow defensiveness was scored as an indicator of the distress caused to the cow by the presence of a handler in the pen due to the potential for it to affect subsequent responses of the cow towards calf pain. During the period from handler entry to exit, the response of the cow was scored according to the method of [17] using a 6 point scale to capture the position of the cow relative to the handler (where 1 = cow stays >10 m from handler; 6 = cow makes rough contact with handler who is pushed off balance), a 4 point scale to capture the speed of movement of the cow (where 1 = no movement; 4 = vigorous and erratic movement at a pace greater than walking speed) and the presence (score 1) or absence (score 0) of vocalisations by the cow. The separate scores were summed to give a single score per cow out of a maximum possible score of 11.

#### 2.5.2. Subsequent Response after Exit of the Handler

A range of calf behaviours was recorded by all-occurrence continuous observations using video equipment (Canon Legria HF M52 mounted on a tripod with a wide angle lens) for a period of 20 min before entry of the handler to the pen and for 2 h after their exit (Table 1). A single observer analysed the video footage with intra-observer agreement of >90% for a sample of six video clips. Observations ceased whilst the handler was in the pen and the observer analysing the videos was blind to the behaviour that occurred whilst the handler was in the pen. The behaviours extracted from video are those which have previously been shown to increase greatly from baselines close to zero in response to castration and are putative indicators of pain (body tense, twitch, foot stamp, tail wag; [15,16]). Additional behaviours previously observed after rubber ring castration occurred too infrequently to be used in the present analysis (easing quarters, stretch and head turning to look at the affected body part; [15]). Additional behaviours intended to capture the response to ear tagging (ear twitch, ear scratch, head shake; the latter two used by [18]) were also recorded, although again, ear scratch described by [18] occurred too infrequently to be analysed. The wider level of arousal was captured by recording play, lie with the head up, lie with the head down and standing/walking; however, play was removed due to its scarcity. Finally, suckling was recorded although it differs from the other behaviours in requiring participation from both cow and calf and its expression is therefore a composite of care soliciting and care giving. Several of these more general behaviours directly convey information on the level of arousal of the calf (e.g., standing/walking versus lying with the head down) whilst others (play, suckling) require locomotion to execute and are assumed therefore to represent arousal. Where appropriate, duration as well as frequency of behaviours was recorded as described in Table 1. During these recording periods, human disturbance was minimized as cows were able to see outside of their pen. Humans approached the pen only to a maximum proximity of 3 m in order to check the correct functioning of the video camera.

To increase the efficiency of analysing the videos of cow behaviour, we focused on the time period after handling that encompassed the greatest deviation in calf behaviour compared to prehandling baseline levels. To determine the period of greatest interest, the time course of expression of each calf behaviour over the 2 h posthandling period was plotted to allow visual identification of the peak period of behavioural change. For most calf behaviours, the period from 0 to 30 min posthandling encompassed the major deviation in behaviour compared to prehandling or the time period after 30 min posthandling. A repeated measures analysis of variance confirmed that all traits apart from three differed significantly during the 30 min post-treatment as compared to pretreatment and/or the 30–120 min period post-treatment (Table 2). The three traits that showed no significant variation across the three time windows were dropped from subsequent analyses (frequency and duration of lying with the head up and the frequency of stretching). Cow behaviour during the immediate 30 min posthandling window was subsequently analysed.

Six cow behavioural traits were extracted from videos using all-occurrence recording for 20 min before entry of the handler to the pen and 30 min after their exit. Two of these traits captured all overt behaviours seen to be directed towards the calf (calf check and lick/sniff calf) and the remaining four described the cow’s locomotion and feeding behaviour.

### 2.6. Statistical Analysis

All analyses were performed in Genstat 16th edition (VSN International Ltd.), *p* < 0.05 was regarded as the statistically significant threshold and means with standard deviation are presented where appropriate. Seven of the 58 calves were not scored for their immediate response to ear tagging and castration whilst the handler was in the pen as the observer had a poor view. Due to the scarcity of scores 2 and 3 for the calf’s immediate response, the scores were converted into a binary classification whereby scores of 0 and 1 were reclassified as score 0, and scores 2 and 3 were reclassified as score 1. A chi-squared test determined whether males and females differed in their likelihood of scoring 0 or 1.

Five behavioural traits recorded from the video footage (duration of suckling, frequency of the calf lying with the head down; duration of cow feeding; frequency of licking/sniffing the calf; frequency of cow walking) were removed from subsequent analysis as they were highly correlated (r > 0.7) with other traits. Considering that both the frequency and duration of state behaviours were candidates for analysis, this reduced the ethogram in Table 1 to the 10 calf and six cow behavioural traits listed in Table 2.

The per-minute frequency or duration of each cow and calf behavioural trait before handling was subtracted from the expression posthandling to quantify the change in expression resulting from the procedure performed. Approximation to the normal distribution was assessed for these behavioural responses using the Anderson–Darling test. Calf ear twitch and leg twitch were log transformed. A paired *t*-test was used to confirm that the per-minute expression of behaviour of the cow differed between the 20 min pre- and 30 min posthandling periods.

A principle component analysis was performed to create latent variables summarising the 10 calf behavioural traits after standardisation such that each trait had a mean of 0 and standard deviation of 1. Thereafter the effect of the latent variables summarising the calf posthandling behaviour on the change in cow behaviour was assessed using a restricted maximum likelihood model (REML) with each cow behavioural trait used as a separate response variable. Fixed effects were cow age, calf sex, calf sire breed, calf immediate response to handling, calf scores on the first two principle components, cow defensiveness and first-order interactions between principle components 1 and 2 and cow defensiveness. The random model included age at tagging, ambient temperature and calf weight. Main effects that were not statistically significant at *p* < 0.05 were stepwise removed from the model. A REML model was also used to estimate the effect of calf sex on principal component scores with calf sire as a fixed effect and calf weight as a random effect.

## 3. Results

### 3.1. Calf Behaviour

Twenty-eight of the calves scored 0 and 23 scored 1 on the binary scale for their initial response to insertion of the ear tags and rubber ring. Males and females did not differ in their likelihood of scoring 0 or 1 (X^2^ = 0.02, *p* = 0.88).

There was wide variation in each of the 10 calf behaviour traits (Table 2). The frequency of foot stamping, tail wagging, ear twitching and head shaking and the frequency and duration of standing/walking all increased after handling, whilst the duration of lying with the head down decreased (all *p* < 0.001). The first two principal components (PC) of the PCA explained 30.0% and 18.1% of the total variance in the change in behaviour after handling compared to that before. Calves that scored highly on PC1 showed, in order of the loadings of the behaviours on the dimension, a large amount of standing/walking, tail wagging, head shaking and suckling (Table 3). Those that scored highly on PC2, again in order of the loadings on the dimension, showed a large amount of leg twitching, little suckling, and a large amount of foot stamping, tensing of the body, ear twitching and lying. The loading of activity on PC1 suggests that it relates mostly to restlessness or arousal but includes some behaviours indicative of an active pain response (tail wagging and head shaking). In contrast, PC2 was mostly associated with reduced activity and the expression of passive indicators of pain (tensing of the body) but also a suite of active pain indicators (leg twitching, foot stamping, ear twitching). No effect of calf sex on PC1 score was found, whilst males scored more highly on PC2 (F_1,28_ = 5.37, *p* = 0.028), suggesting that the behaviours captured by PC2 related mostly to castration.

### 3.2. Effect of Calf Behaviour on Cow Behaviour

The summed defensiveness score of the cows whilst the handler was inside the pen was 6.9 (SD 2.12) out of a maximum possible score of 11. The defensiveness score was not significantly related to the increase or decrease in any of the six cow behaviours recorded for 30 min after handling. However, cow behaviours all changed in response to treatment of their calf at a population level (Table 2) with the exception of feeding behaviour of the cow which did not differ between the pre- and post-calf-handling periods.

Calves that showed a greater response to insertion of the ear tags (and rubber ring) by scoring 1 on the binary scale expressing their initial response to the procedures received a lower duration of sniffing and licking from their mother than calves that scored 0 (0.85 vs. 4.68 s/min, F_1,40_ = 5.0, *p* = 0.031). Calf sex did not significantly influence the behaviour shown by the cow, although there was a statistical tendency for male calves to receive more calf check behaviour (0.30 vs. 0.14 events per minute in males and females, F_1,47_ = 3.69, *p* = 0.061). Calves that scored highly on PC1 received more calf checking (F_1,47_ = 20.4; *p* < 0.001), and their mother tended to stand more frequently (F_1,48_ = 3.14, *p* = 0.083) and feed less frequently (F_1,48_ = 4.11, *p* = 0.048). There was no significant relationship between the calf score on PC2 and the behaviour of the cow. Furthermore, the scores on PC1 and PC2 did not interact to significantly affect the cow’s behaviour, nor was there an interaction between the cow’s defensiveness score and either PC1 or PC2 on the cow’s behaviour.

## 4. Discussion

The overall aim of this study was to examine whether cows behaviourally respond to behaviours previously validated as indicators of pain in their calves in addition to their altered arousal or restlessness as a result of management procedures. Cows were generally responsive to the presence of the handler in the pen and calf-directed behaviours shown by the cow approximately doubled following the procedures performed on their calf. The loadings of calf behaviours on principle components 1 and 2 indicate that PC1 related mostly to activity and expression of active indicators of pain and PC2 to reduced activity and heightened expression of both active and passive pain indicators. Cows showed a greater increase in their frequency of checking the calf after handling if it scored highly on PC1, suggesting that they were receptive to heightened activity and some specific active behavioural expressions of pain. However, cows did not differentiate between male and female calves in their level of care despite the males being castrated and showing increased expression of many pain indicators, which was against the prediction in hypothesis 1. Furthermore, considering both calf sexes together, cow behaviour was unaffected by calf score on PC2, contrary to hypothesis 2. This suggests that cows did not alter their care-giving in response to calf lethargy or the active and passive indicators of pain captured by this PC. Regarding the immediate response of the calf to restraint and the handling procedure, it is likely that the behaviour recorded at this time only captured acute pain associated with ear tagging and not pain associated with rubber ring castration which likely occurred after the handler had left the pen. This would be in agreement with earlier work which found that observers were unable to detect an immediate change in behaviour on application of a rubber ring [20]. Nevertheless, an unexpected negative effect on the duration of licking and sniffing of the calf when the calf struggled during the handling procedure confirmed that the cows did not increase their attention towards calves that expressed an acute pain response or an aversion to handling.

We used calves as their own controls and were unable to use a sham-handled treatment. With this design, it is not possible to test whether behavioural changes following the husbandry procedures would also have been seen following sham treatment. However, ear tagging of both sexes and castration of males resulted in increased expression of specific behaviours previously found to be elevated after rubber ring castration and ear tagging but not in response to sham treatment (e.g., [15,18,21]) suggesting that the calves did show behavioural responses that were specifically indicative of pain. Several of these behaviours have been shown to be mitigated by pain relief [22,23]. Furthermore, it was not possible to prevent the cow from viewing the handling of her calf. However, this occurred for all cows and would be expected to heighten, not diminish, her response to subsequent calf behaviour. It was also probably less stressful for the cow than removing her neonatal calf from her sight which might have overshadowed any association between the calf’s behaviour and her response to it.

The first two dimensions of the PCA explained only 48.1% of the total variance in the change in calf behaviour after handling compared to that before. Evidence that males scored more highly on PC2 might indicate that the behaviours captured by this PC reflected the diffuse, visceral and more chronic nature of pain caused by rubber ring castration as compared to more acute and localised pain caused by ear tagging. A number of behaviours reported previously to increase in response to ear tagging (ear scratch in pigs; [18]) and rubber ring castration (easing quarters, stretch and head turning of calves; [15]) occurred very rarely or not at all in the present population throughout the whole 2 h posthandling observation period and hence were not analysed. The space afforded to the calves, freedom from disturbance during the observation period and quality of video footage were sufficient to allow these behaviours to have been detected had they occurred. The use of a single observer was preferred as it prevented interobserver error. However, it is not possible to estimate how observations made by this observer might have compared to that of other people. Nonetheless, calves in the present study stood or walked for only 17 s per minute after handling, indicating that the activity level was generally low. However, the increased frequency of standing and walking after handling suggests that more frequent transitions in posture occurred. These animals were handled within the first day of life, which is substantially earlier than previous studies examining behavioural responses to castration (e.g., 5–7 days, [15]; 3–4 weeks, [16]; 4–6 weeks, [21]; 6–25 months, [20,24]). Robertson et al. (1994) [25] and Boesch et al. (2008) [19] studied the response of calves to castration within the first week of life and similarly found that some of the behavioural changes reported in older calves were rare or absent at this age, although evidence of pain was generally present. Additionally, Meléndez et al. (2017) showed that the physiological response and duration of behavioural response were less in calves castrated at 1 week as compared to 2 or 4 months of age [26]. We chose an early age as this represents the time window when calves are conventionally handled in the UK before being rapidly moved to pasture and where domestic legislation requires that rubber ring castration be performed within the first 7 days of life. Furthermore, we expected that maternal care would be particularly focused during this highly vulnerable period as defensiveness shown by beef cows peaks immediately after calving before declining rapidly and cows tend to direct greatest attention towards small and vulnerable calves [17,27,28,29]. However, neuroendocrine and electroencephalographic responses to castration have been shown to increase with calf age [30]. It is possible that behavioural responses are also age-dependent and that those of neonatal calves were too subtle to be detected by the cow. Cows in natural environments typically hide their neonatal calves whilst they forage [31], and low calf activity may have evolved as a response to predation risk. This scenario may also have reduced selection pressure for dams to be responsive to neonatal calf behaviour. Although we focussed only on cows which had calved without difficulty or assistance, it is also possible that her recent experience of the stress of calving interfered with the quality of care the cow directed towards the calf.

As measures of maternal care we recorded sniffing, licking, suckling and brief orientation towards the calf accompanied by tactile contact (‘calf check’). Whilst we expected that this would adequately capture the cow’s interactions with her calf, other more subtle behaviours were not included, such as glances as used by [13] or orientation without tactile contact used by [14]. However, the behaviours we recorded are those that required the cow to invest effort in caring for the calf and have previously been used to characterise maternal behaviour in cattle (e.g., [31,32]). The evidence from the present study suggests that, had cows been receptive to the level of pain expression of their calf, this was not manifest in alterations to these key indicators of maternal interaction.

Contrary to hypothesis 1, cows did not show greater attention towards male calves despite these being castrated. Other work suggests that male beef calves receive greater maternal protection than female calves between the ages of 3 and 30 days [29], although this does not appear to extend to greater expression of behaviours such as licking or suckling [32]. The 30 min posthandling period when observations were performed coincided with the peak in expression of putative pain behaviours in our population during a recording window up until 2 h posthandling. This is slightly earlier than the 1 h delay until peak behavioural expression in older calves in the study of [16] but corresponds to the latency until peak plasma cortisol response reported by [15,16,20,21]. A 30 min period was also adequate to detect significant changes in ewe responses to rubber ring castration of their lambs [14]. It therefore seems unlikely that our observation window was too short to detect a heightened response of the cow to the castration of male calves had such a response occurred.

The score that cows received on the defensiveness scale whilst the handler was in the pen did not affect any of the six cow behavioural traits measured in the 30 min after the handler left the pen. This is in agreement with [17] that maternal defence is independent from other maternal behavioural traits and fits with data from other species where maternal behaviour can be experimentally induced in isolation from maternal defensive aggression (rats, [33]; mice, [34]). Furthermore, the cow’s behaviour after the handler left the pen was not significantly affected by the interaction between her defensiveness score and the score of her calf on PC1 or PC2, suggesting that the sensitivity of a cow to the behaviour of her calf was independent of her defensiveness.

## 5. Conclusions

Our results indicate that some aspects of cow maternal behaviour are responsive to the level of arousal of the calf coupled with expression of specific behaviours indicative of an active pain response after management procedures performed on the first day of life. However, cows appear not to alter their maternal behaviour according to the expression of passive nor the majority of active behaviours shown in earlier studies to be indicative of pain from ear tagging and rubber ring castration. Furthermore, despite male calves scoring more highly on PC2, probably as a result of castration, cows did not differentiate between the sexes in their maternal care behaviour. Whilst on the one hand the vulnerability of a neonatal calf and its need for maternal protection might be expected to enhance maternal responsiveness to calf distress, this may have been overshadowed by the recent physical and emotional stress of calving and the general inactivity of calves of this age. It would be interesting to test whether a greater maternal response is evident in slightly older calves but, under the scenario tested, little behavioural evidence was found that cows alter their maternal care in response to the behavioural expression of their calf following management interventions that are assumed to be painful.

## Figures and Tables

**Table 1:** Ethogram of behaviours recorded by continuous observations for 20 min before entry of the handler to the pen and for 2 h after handler exit, of which the first 30 min were used in subsequent analyses. The third column states whether the behaviour was analysed based upon its frequency, duration or both. Definition sources are cited unless the definition was created by the authors.

Calf Event Behaviours		Analysed as:
Body tense	Stiffening of the whole body while lying.	Frequency
Leg twitch	Sudden twitching or kicking out of hind limb while lying [19].	Frequency
Foot stamp	Lifting and forcefully placing hind limb on ground while standing [16].	Frequency
Tail wag	Tail movement from side to side or in a continuous series recorded as a single event [15]. Event ends when tail returns to normal vertical hanging position.	Frequency
Ear twitch	Rapid, seemingly uncontrolled flicking movement of ear without vigorous movement of the rest of the head.	Frequency
Head shake	Vigorous toss of the head or shaking of the ears [18].	Frequency
**Calf state behaviours**		
Lie head down	Lying with weight of head supported primarily by the ground or resting on the animal’s own body.	Duration
Lie head up	Lying with weight of the head supported by the neck.	Not analysed
Stand/walk	Body weight supported by all four legs and animal either stationary or moving.	Frequency and duration
Suckle	Calf suckles dam or seeks a teat while cow is standing. Teat seeking behaviour comprises orientating head anywhere around the dam’s lower body while touching her body with nose/mouth.	Frequency
**Cow event behaviours**		
Calf check	Brief orientation towards the calf and tactile contact with any part of its body lasting less than 2 s.	Frequency
**Cow state behaviours**		
Lick/sniff calf	Standing (as described below) while performing a bout of licking/sniffing any part of the calf. During sniffing the nose is within approximately 0.5 m distance from calf and cow is seen to actively reach forward and investigate the calf. Close proximity of the nose to the calf alone does not classify as sniffing.	Duration
Stand	Standing stationary on all four legs or moving in a manner not classified as walking as defined below. Locomotion in a backwards direction, shifting weight and re-orientating position are all classified as standing. Movement is usually a series of small steps, pauses and lacks a defined purpose, direction and flow.	Frequency and duration
Walk	Forward locomotion resulting in the animal moving a minimum distance of around one body length.	Duration
Feed	Head is on the opposite side of the feed barrier to the body and vertically above the feed area.	Frequency

**Table 2:** Descriptive statistics of the mean frequency and duration per minute of calf and cow behaviours during the 20 min before calf handling and 30 min after handling.

Behaviour	Before Calf Handling		After Calf Handling		SED	Significance
	Mean	SD	Mean	SD		
**Calf behaviours**						
Body tense (n/min)	0.087	0.129	0.077	0.117	0.019	NS
Leg twitch (n/min)	0.084	0.152	0.093	0.167	0.029	NS
Foot stamp (n/min)	0.011	0.039	0.092	0.182	0.018	<0.001
Tail wag (n/min)	0.228	0.650	0.621	0.653	0.093	<0.001
Ear twitch (n/min)	0.068	0.083	0.320	0.220	0.027	<0.001
Head shake (n/min)	0.084	0.110	0.519	0.403	0.043	<0.001
Lie head down (s/min)	16.18	18.01	5.04	6.91	2.202	<0.001
Stand/walk (n/min)	0.112	0.148	0.251	0.174	0.024	<0.001
Stand/walk (s/min)	6.12	8.50	17.17	11.21	1.466	<0.001
Suckle (n/min)	0.035	0.102	0.068	0.133	0.017	NS
**Cow behaviours**						
Calf check (n/min)	0.18	0.178	0.42	0.279	0.040	<0.001
Lick/sniff calf (s/min)	3.39	6.307	6.43	7.292	0.912	=0.001
Stand (n/min)	0.59	0.455	0.99	0.383	0.064	<0.001
Stand (s/min)	36.4	17.13	42.9	10.53	2.05	<0.01
Walk (s/min)	1.58	2.333	2.40	2.762	0.356	<0.05
Feed (n/min)	0.13	0.203	0.15	0.177	0.031	NS

**Table 3:** Loadings of each of the 10 calf behavioural traits on principal components (PC) 1 and 2.

Calf Behaviour	Loading on PC1	Loading on PC2
Body tense (n/min)	−0.11	0.32
Leg twitch (n/min)	0.06	0.58
Foot stamp (n/min)	0.22	0.39
Tail wag (n/min)	0.38	−0.12
Ear twitch (n/min)	0.16	0.26
Head shake (n/min)	0.38	0.22
Lie head down (s/min)	−0.30	0.24
Stand/walk (n/min)	0.49	−0.09
Stand/walk (s/min)	0.49	0.17
Suckle (n/min)	0.28	−0.43
